# Implementation of mobile health interventions in hypertension management and outcomes: A scoping review protocol

**DOI:** 10.1371/journal.pone.0342224

**Published:** 2026-02-05

**Authors:** Saviour Edem Vidzro, Monica Ansu-Mensah, Siyabonga Dlamini, Desmond Kuupiel

**Affiliations:** 1 Discipline of Public Health Medicine, School of Nursing and Public Health, University of KwaZulu-Natal, Howard College Campus, Durban, South Africa; 2 Institute of Health Research, University of Health and Allied Sciences, Ho, Ghana; 3 Directorate of Grants, Research and Development, Sunyani Technical University, Sunyani, Bono Region, Ghana; 4 Cancer and Infectious Diseases Epidemiology Research Unit, College of Health Sciences, University of KwaZulu-Natal, Howard College Campus, Durban, South Africa; 5 Department of Nursing and Public Health, Faculty of Health Sciences, University of Fort Hare, East London, South Africa; The Hong Kong Polytechnic University, HONG KONG

## Abstract

**Background:**

Hypertension remains a global health concern, significantly increasing the risk of cardiovascular disease, stroke, and mortality. Mobile health (mHealth) interventions are emerging as a promising solution for hypertension management, offering tools for remote monitoring, patient engagement, and data tracking. This scoping review aims to map the existing literature on mHealth interventions for hypertension, highlighting key trends, research gaps, and implications for clinical practice and future research.

**Objective:**

This scoping review aims to map the breadth of literature on mHealth interventions for hypertension management, focusing on implementation (feasibility, acceptability, adoption), effectiveness, and health outcomes. Specifically, it seeks to identify the types of mHealth interventions used, implementation strategies across various settings, barriers and facilitators, and associated health outcomes.

**Methods:**

This review will consist of a systematic literature search of databases such as PubMed, Scopus, Cochrane Library, Google Scholar, Web of Science, EBSCOhost, and EMBASE, augmented with grey literature, in accordance with the methodological framework developed by Arksey and O’Malley and improved by Levac et al. The study will be chosen using a two-phase screening procedure, and the Mixed Methods Appraisal Tool (MMAT) will be used to evaluate the study’s quality. Study characteristics, population information, intervention descriptions, results, and important findings will all be captured during data extraction.

**Results:**

The review will identify the types of mHealth interventions, assess their effectiveness in blood pressure control, and evaluate key implementation outcomes, including feasibility, acceptability, and adoption as reflected by patient adherence, engagement, and satisfaction. Additionally, key research gaps will be highlighted, such as the need for long-term studies and diverse population representation.

**Conclusion:**

This scoping review will provide a comprehensive overview of mHealth interventions for hypertension management, identifying effective strategies and highlighting areas for further research. The findings will inform healthcare providers, researchers, and policymakers on integrating mHealth technologies into hypertension care to improve patient outcomes.

## Background

Hypertension, also known as high or raised blood pressure, is one of the most prevalent chronic diseases worldwide and is a significant contributor to cardiovascular (CV) mortality [1]. The World Health Organization (WHO) defines hypertension as a condition in which blood vessels have persistently raised pressure, typically defined as systolic blood pressure (SBP) greater than or equal to 140 mmHg and diastolic blood pressure (DBP) greater than or equal to 90 mmHg [1,2]. Globally, hypertension affects more than 1.2 billion adults aged 30–79 years, with two-thirds residing in low-and-middle-income countries [1]. Alarmingly, about 46% of these individuals are unaware of their condition, highlighting a significant gap in hypertension management [3].

In Africa, the burden of hypertension is substantial, with an estimated 74.7 million individuals affected [4], the national pooled prevalence of hypertension in Ghana is approximately 30.3% (95% CI: 26.1–34.8%) [5]. The prevalence of hypertension is rising due to urbanization, lifestyle changes, and increasing risk factors such as obesity and sedentary behaviour [6]. However, awareness and control remain low, with many individuals undiagnosed and untreated [3]. In sub-Saharan Africa and other low- and middle-income countries, hypertension prevalence continues to rise, with recent estimates indicating prevalence rates of approximately 28–30% among adults in several countries, including Ghana. Despite this high burden, hypertension control rates remain low due to limitations in health system capacity, access to care, and continuity of long-term management. These contextual challenges underscore the relevance of scalable and resource-efficient interventions such as mHealth in low-resource settings [7]. The WHO emphasizes the need for innovative solutions to improve awareness, treatment, and control of hypertension, particularly in underserved populations [8].

mHealth technologies which include mobile applications and telemedicine, have emerged as promising tools for addressing various health challenges, including chronic disease management like hypertension. mHealth involves the use of mobile devices such as smartphones, tablets, and wearable sensors to support medical and public health practices [9]. The ubiquity of mobile technologies has opened new avenues for patient engagement, real-time monitoring, and personalized health management strategies, making it a cost-effective and accessible approach, especially in resource-constrained settings [10].

For hypertension management, mHealth interventions include applications that provide medication reminders, promote lifestyle modifications, track blood pressure readings, and facilitate communication between patients and healthcare providers [11]. Such interventions have the potential to improve adherence to antihypertensive medications, encourage healthy behaviours, and enhance patient-provider interactions, leading to better blood pressure control and health outcomes [12].

Studies have shown that mHealth interventions can lead to significant reductions in blood pressure and improve overall health outcomes [13]. For instance, several studies have indicated that mHealth applications could effectively support self-management of hypertension by providing reminders for medication, educational resources, and direct communication with healthcare providers [14,15]. Similarly, a randomized controlled trial in Nigeria and Ghana demonstrated that mHealth interventions significantly reduced systolic blood pressure among hypertensive patients, suggesting that such technologies can play a crucial role in managing hypertension in sub-Saharan Africa [14]. However, despite the growing interest in mHealth solutions for hypertension, the implementation of these interventions and their associated outcomes vary across different settings and populations.

To date, there is limited comprehensive scoping review synthesis of the evidence surrounding the implementation of mHealth interventions specifically targeting hypertension management. A search in the literature shows several systematic reviews and meta-analysis on mHealth application for hypertension, and a few scoping reviews [16–18]. Scoping reviews are essential to map the breadth and scope of available literature on a broader topic, identifying key concepts, gaps, and types of evidence [19]. Although existing systematic and scoping reviews of mHealth interventions for hypertension largely focus on effectiveness and clinical outcomes, evidence on implementation processes, context, feasibility, and sustainability remains limited. This scoping review addresses this gap by mapping implementation-related evidence alongside effectiveness to identify intervention types, strategies, barriers, and facilitators, and to inform future research, policy, and scalable hypertension management practice.

## Method

This scoping review is part of a PhD study and follows Arksey and O’Malley’s methodological framework, as refined by Levac et al. [19], to provide a systematic and comprehensive mapping of the literature. The framework involves five key stages: i) identifying the research questions, ii) identifying the relevant studies, iii) selecting relevant studies, iv) charting the data, collating, summarizing, and v) reporting the results/findings. This approach will ensure a systematic mapping of the available evidence on the implementation of mHealth interventions for hypertension management and outcomes. We will use Covidence for article screening, import and de-duplicate citations using EndNote, manage and export data via Excel or statistical software, and conduct qualitative analysis using NVivo, thereby covering all major data-management and review-analysis phases. The scoping review will be conducted over an extended and phased timeline to ensure methodological rigor. Title and abstract screening will be undertaken over approximately 6–8 weeks, followed by full-text screening over 4–6 weeks. Data extraction and quality appraisal will be conducted concurrently over a further 6–8 weeks. Data synthesis and reporting will then be completed over 4–6 weeks. The review process is expected to span approximately 4–6 months, which is considered realistic given the anticipated volume of records and the complexity of screening, extraction, and synthesis.

No formal ethics approval was required because the study used only publicly available, published literature.

### Step 1: Identifying the research question

The main research question: What evidence exists on the implementation of mHealth interventions for hypertension management and their associated health outcomes? In this review, “implementation” is conceptualised using an implementation science perspective. Specifically, the review focuses on implementation outcomes such as feasibility, acceptability, adoption, fidelity, sustainability, and scalability of mHealth interventions for hypertension management, rather than merely describing the existence of implemented interventions.

Studies involving participants with multiple conditions (e.g., diabetes and hypertension) will be included if the mHealth intervention explicitly targets hypertension management as a primary objective. Studies will be excluded if hypertension is only mentioned as a background or secondary condition and the mHealth intervention primarily targets another disease or health outcome. The eligibility criteria for study inclusion and exclusion are summarised in (S1 Table).

Sub-questions include:

1. What types of mHealth interventions or tools have been implemented to manage hypertension?
2. What strategies have been used to implement these interventions across different settings?
3. What are the reported facilitators and challenges and/or barriers to the implementation of mHealth interventions for hypertension management?
4. What health outcomes (e.g., blood pressure control, medication adherence, and lifestyle changes) have been associated with the implementation of these interventions?

### Step 2: Identification of the relevant studies

We will perform a thorough literature search using a variety of databases and sources in order to fully compile the body of knowledge regarding mHealth interventions for the management of hypertension. MEDLINE (PubMed), the Cochrane Library, Scopus, Google Scholar, Web of Science, EBSCOhost (PsycINFO and CINAHL), and EMBASE are among the key databases that we will search. Because of their thorough coverage of the medical, psychological, and healthcare literature, these databases will be chosen to ensure that our review includes a broad variety of pertinent publications on mHealth therapies for hypertension.

To ensure a comprehensive and inclusive search, grey literature sources will be reviewed in addition to these core databases. Grey literature sources will be searched to identify additional relevant peer-reviewed studies and contextual information; however, only peer-reviewed journal articles will be included in the final synthesis. In addition to bibliographic databases, selected organizational websites and repositories will be searched to identify relevant peer-reviewed publications and contextual materials. These will include the World Health Organization (WHO), the Centers for Disease Control and Prevention (CDC), the Global Digital Health Network, the World Bank, and national Ministry of Health websites in low- and middle-income countries. Conference proceedings and publication repositories of major digital health and cardiovascular forums, such as the Global Digital Health Forum, mHealth Summit, World Congress of Cardiology, and International Society of Hypertension, will also be searched to identify eligible peer-reviewed outputs, country-level institutional repositories for Ghana and sub-Saharan Africa, and conference portals for major digital health and hypertension. This method guarantees that the review includes all of the available data on mHealth technology in the treatment of hypertension and helps to reduce publication bias.

To strengthen transparency and reproducibility, we will include the complete, finalized search strategies for all databases in the protocol’s appendix. These will show exact Boolean strings, search fields, limits, and dates of execution, in line with reporting best-practice standards such as the PRISMAP checklist. The search phrases will cover important ideas that are essential to this review, such as wearable technology, telehealth, mHealth, mobile applications, hypertension, and text messaging.

Finding research on hypertension will be made easier with the inclusion of the main search keyword, “hypertension.” A wide range of digital health tools will be covered by terms like “mobile health,” “mHealth,” “mobile applications,” “telehealth,” “text messaging,” and “wearable devices.” To filter studies that address the particular context of interest, these terms will be coupled with “hypertension management”. The search will be narrowed using Boolean operators (AND, OR), which will include studies that satisfy the inclusion criteria and omit those that don’t.

For each database, we will adapt the search approach to take use of its distinct features and indexing methods. Customizing keywords and MeSH terms to match the thesaurus and indexing terms of each database will be part of this adaption.

The search strategy was developed in consultation with an academic librarian. The finalized PubMed search strategy is presented within the main text, and equivalent database-specific adaptations will be applied for other databases (e.g., Scopus, Web of Science, EMBASE, and Cochrane Library) by modifying controlled vocabulary and indexing terms while retaining the same conceptual structure and Boolean logic. Full search details, including dates of execution and limits applied, will be reported in the final review. The keywords for the search are to include terms such as “hypertension,” “mHealth,” “blood pressure control,” “self-management,” “patient engagement,” and “intervention effectiveness.” These terms will be searched as both keywords in titles, abstracts, and/or subject headings (e.g., MeSH terms) to capture relevant studies. Retrieved articles will undergo screening based on titles, abstracts, and indexed terms. All records retrieved from each database will be imported into EndNote, a reference management software, which will aid in record management, article tracking, and compiling a reference list for the final review report. Following evaluation of pilot searches, search #2, #6 and #10 have been selected as the primary base strategies for finalisation, and will be adapted for other databases with additional limits (publication date, language, LMIC context). S2 Table summarises preliminary search strings tested in PubMed, including keywords, Boolean operators, search dates, and the number of records retrieved for each strategy.

### Step 3: Selection of eligible studies

There will be two screening stages during the article evaluation process. First, two reviewers SEV (PhD candidate in Public Health with training in systematic and scoping review methodology and digital health research) and MAM (PhD, with expertise in evidence synthesis and health systems research) will independently screen titles and abstracts of all retrieved citations against minimal inclusion criteria. Second, the full texts of citations deemed potentially relevant by one or both reviewers will be retrieved and evaluated in full. To ensure consistent application of the eligibility criteria, the two reviewers will independently screen records. A third reviewer will be selected from a pool of senior faculty members in the Institute of Health Research, who have expertise in digital-health evaluation but were not involved in developing the protocol. The third reviewer will be engaged to adjudicate any disagreements between the first two reviewers, ensuring an independent assessment. Before full screening, a pilot screening exercise will be conducted on a random sample of approximately 10% of retrieved records. This pilot phase will be used to refine the eligibility criteria, ensure consistent interpretation between reviewers, and calibrate the screening process. Any ambiguities identified during the pilot screening will be discussed and resolved before proceeding to full screening. Article screening will be conducted using Covidence systematic review software to facilitate independent title/abstract and full-text screening, record management, and resolution of reviewer disagreements. EndNote will be used for reference management and removal of duplicate records prior to screening.

Inter-rater reliability between the two reviewers will be assessed using Cohen’s kappa statistic (κ). A kappa value of κ ≥ 0.60 will be considered indicative of substantial agreement. Discrepancies will be discussed and resolved through consensus, with involvement of a third reviewer when necessary [20].

After that, a team member will review the article titles and abstracts to pick out those that don’t fit the eligibility requirements specified in the protocol’s second step. The titles and/or abstracts that satisfy the inclusion requirements will be used to retrieve the full-text publications.

Until a consensus is reached, or by the determination of a third reviewer, if necessary, disagreements regarding the study eligibility of the sampled articles will be considered between the two reviewers. The selection of studies for this review will be reported using the Preferred Reporting Items for Systematic Reviews and Meta-Analyses (PRISMA) flow chart (S1 Fig) [21], which outlines the detailed search, screening, eligibility assessment, and inclusion process.

### Quality assessment

The Mixed Methods Appraisal Tool (MMAT), Version 2018, will be used to evaluate the quality of the research that are part of this scoping review. By taking into account elements including research relevancy, design, methodological sufficiency, data collection procedures, data analysis, and conclusions, this tool will assess each study’s methodological rigor. While no studies will be excluded based on methodological quality (as is typical for a scoping review), we will conduct a basic appraisal of study quality/risk of bias and use these findings to contextualize our synthesis, highlighting how methodological limitations may affect interpretation and mapping of evidence.

Consistent with scoping review methodology, methodological quality will not be used as an exclusion criterion. Instead, findings from the quality appraisal will be used to contextualise the synthesis by highlighting common methodological strengths and limitations across the included studies. Quality considerations will be discussed narratively alongside the results to aid interpretation of the evidence base, rather than to assess risk of bias at the individual study level or to weight study findings.

We will use the Mixed Methods Appraisal Tool (MMAT) for a qualitative description of methodological quality rather than calculating a composite percentage score. Each study will be assessed against MMAT’s criteria and classified using qualitative, based on the pattern of criteria met. The quality assessment will inform the interpretation and discussion of findings, including identifying potential bias or areas of methodological weakness, but will not be used to exclude studies or generate numerical ranking. MMAT will be treated independently in extraction and synthesis, with results clearly reported and integration issues discussed.

Since the goal of this scoping review is to map the body of existing literature thoroughly, the quality appraisal will not be used as an exclusion criterion. But by evaluating quality, we may spot potential weak points in the research, point out gaps in solid data, and provide suggestions for more research. To promote openness and aid in a better comprehension of the advantages and disadvantages of the included research, the quality evaluation results will be displayed in a summary table.

### Step 4: Charting the data

Data charting form will be developed to facilitate the extraction of relevant data from the studies included in this scoping review on hypertension management using mHealth interventions. The data charting form would include implementation science framework domains (RE-AIM and CFIR), thus the Reach, Effectiveness, Adoption, Implementation, and Maintenance (RE-AIM) framework and key domains of the Consolidated Framework for Implementation Research (CFIR). These frameworks will guide the extraction of implementation-related data, intervention characteristics, inner and outer setting factors, implementation processes, and contextual barriers and facilitators (CFIR).

1. Study Characteristics: Details including author names, publication year, country of origin, and study design.
2. Studies employing multiple designs (e.g., randomized controlled trials with embedded qualitative components or mixed-methods studies) will be treated as single studies and classified according to their overall study design. Data from each methodological component will be extracted and charted separately where relevant (e.g., quantitative outcomes and qualitative implementation insights) to ensure that all relevant evidence is captured without double-counting.
3. Population Details: Information on participant demographics, sample size, and any hypertension-specific characteristics (e.g., severity, comorbidities).
4. Description of mHealth Intervention: Type of digital technology used (e.g., mobile app, SMS-based reminders and wearable devises), key features and functionalities (such as blood pressure monitoring, medication reminders), and the intended user group (e.g., hypertensive patients, healthcare providers).
5. Intervention duration and intensity – We will capture the total length of exposure (e.g., number of months the app was used), session frequency or reminder frequency, and overall dose of the intervention. Evidence shows that both duration and dose can significantly moderate outcomes.
6. Theoretical frameworks used – We will chart which behaviour change or implementation science theories (e.g., Health Belief Model, Technology Acceptance Model, COM-B model) underpin each intervention, thereby enabling analysis of whether theory-guided apps show differential implementation or outcome success.
7. Cost or resource requirements – We will include data on resource use (devices, data costs, training time, staffing) and cost-effectiveness when available, recognizing that feasibility and scalability depend on resource intensity.
8. Equity considerations – We will record whether each study reports on access by socio-economic, age, gender, digital literacy or rural/urban subgroups, to analyse whether mHealth apps are reaching underserved groups.
9. Data security/privacy measures – We will document whether interventions included features such as encryption, data minimisation, authentication, user consent modules or regulatory compliance, given the rising concerns about data privacy in mHealth.
10. Outcomes Measured: Primary and secondary outcomes such as changes in blood pressure, adherence to medication, self-monitoring practices, user satisfaction, and continuity of care.
11. Key Findings and Conclusions: Insights into implementation strategies, effectiveness, user satisfaction, policy and guideline usability, as well as ethical, legal, or social considerations. Barriers, challenges, and facilitators relevant to mHealth use in hypertension management will also be documented.

The data extraction form will be refined as needed based on feedback from two independent reviewers to ensure it effectively captures all necessary data for addressing the research question. To maintain consistency and accuracy, the form will be piloted by the reviewers on a random sample of 10% of the included studies. This piloting process will help identify any necessary adjustments to the extraction form, ensuring it is comprehensive and suitable for the scope of this review. We will integrate a pilot screening phase using a small sample of citations to calibrate eligibility criteria and reviewer alignment before full screening begins.

### Step 5: Collating, summarising, and reporting the results

The findings of this scoping review will be collated, summarized, and reported using a structured approach based on the framework established by Arksey and O’Malley recommended by Levac et al. but for this scoping review, we will not include the optional sixth stage (“stakeholder consultation/knowledge-user engagement”) methodological framework, since our focus is on mapping published evidence rather than engaging external stakeholders [19]. Although Arksey and O’Malley’s framework includes an optional sixth stage involving stakeholder consultation, this stage will not be undertaken in the present scoping review. The primary aim of this review is to systematically map and synthesise published evidence on mHealth interventions for hypertension management. Stakeholder engagement with patients, clinicians, and policymakers is planned for subsequent phases of the broader PhD project, where findings from this scoping review will inform intervention design, implementation, and contextual adaptation.

Initially, all extracted data will be systematically organized to facilitate analysis. This will include creating a descriptive summary of key study characteristics such as authorship, publication year, country of origin, study design, population, and the type of mHealth interventions examined. Studies will be grouped according to thematic categories identified during data extraction to provide an overview of the research landscape related to hypertension management through mHealth interventions.

Data summarization will encompass both quantitative and qualitative analyses. Quantitative summaries will involve basic descriptive statistics, including the number of studies categorized by study type, geographic location, patient population, and intervention types. This approach will help to outline the scope and nature of the existing literature. Qualitative synthesis (thematic analysis procedures): We will use a hybrid inductive-deductive thematic analysis: initial codes will be informed by theory and prior research, while remaining open to new themes emerging from the data; two independent coders will conduct the analysis using NVivo software, and methodological rigor will be ensured through reflexivity journals, peer debriefing, and transparent reporting of analytic decisions as described by Braun and Clarke [22]. This will include examining trends in mHealth technologies, implementation barriers, facilitators of successful interventions, and reported outcomes related to hypertension control and patient engagement [22,23]. The review will also assess temporal trends by grouping studies by publication period to examine how mHealth interventions for hypertension, including intervention types, implementation strategies, and outcomes, have evolved over time in response to technological advances and changing healthcare contexts.

The final report of the scoping review will adhere to the PRISMA-P guidelines, which provide a standardized framework for reporting scoping reviews [21]. The results will be presented in a narrative format supported by tables and figures to illustrate the distribution and characteristics of the included studies. Additionally, the review will highlight gaps in the current literature, offering insights into areas needing further investigation. The discussion will address how the findings enhance understanding of mHealth interventions in hypertension management and their implications for clinical practice, policy, and future research.

## Discussion

This scoping review aims to provide a comprehensive mapping of the existing literature on mHealth interventions for hypertension management. By systematically identifying and analysing the range of interventions, study designs, and outcomes [19] the review will contribute to a deeper understanding of how mHealth technologies are used to manage hypertension. This understanding is essential for healthcare professionals, researchers, and policymakers to identify effective strategies and inform the development of new interventions [21].

The review’s findings are expected to have a significant effect in several fronts. For clinicians, it will offer insights into the current state of mHealth interventions and their effectiveness in managing hypertension. By highlighting successful interventions and common features, the review may guide clinical practice and support the integration of mHealth technologies into routine hypertension care [24]. For researchers, the review will identify gaps in the current literature and suggest areas for further investigation, potentially leading to more targeted research efforts and the development of new, innovative mHealth solutions [25].

One of the key contributions of this scoping review will be to highlight gaps in the existing literature and suggest directions for future research. For instance, if the review identifies a lack of studies on the long-term effects of mHealth interventions, this will signal a need for longitudinal research to evaluate the sustainability of these interventions [26]. Additionally, if the review reveals a gap in research involving diverse populations, it will highlight the importance of including underrepresented groups in future studies to ensure that mHealth interventions are broadly applicable and equitable [27]. Although several systematic reviews and meta-analyses have examined mHealth interventions for hypertension, particularly in relation to engagement, usability, and clinical efficacy, such reviews have largely focused on app features and user engagement. For example, a systematic review by Cao et al. explored engagement and interactivity in mHealth apps for hypertension self-management but did not comprehensively examine broader implementation outcomes such as adoption contexts, sustainability, equity, cost, and scalability, which are central to the present scoping review [28].

Furthermore, the review will provide a foundation for developing standardized frameworks for evaluating mHealth interventions. This can facilitate the comparison of different studies and the establishment of best practices for the design, implementation, and assessment of mHealth technologies in hypertension management [21,22]. Due to language barriers and limitations in translation resources, this review will include only studies published in English; we acknowledge that this may lead to the omission of relevant evidence published in other languages and may introduce language-related bias.

## Conclusion

In conclusion, this scoping review protocol aims to systematically map the literature on mHealth interventions for hypertension management. Through a comprehensive search and rigorous selection process, the review will provide insights into the effectiveness, challenges, and opportunities of integrating mHealth tools into hypertension care. The findings will guide future research, policy-making, and clinical practice, supporting the development of innovative and scalable solutions to improve hypertension management, particularly in resource-limited settings.

## Supporting information

S1 Table Study eligibility criteria used for selecting articles in the scoping review. (DOCX)

S2 Table PubMed database pilot search strategy used to inform development of the final search strategy for the scoping review. (DOCX)

S3 Table PRISMA-P (Preferred Reporting Items for Systematic Review and Meta-Analysis Protocols) 2015 checklist for the scoping review protocol. The table summarises compliance with PRISMA-P reporting items and indicates where each item is addressed in the manuscript; items not applicable to scoping reviews are noted.
(DOCX)

S1 Fig PRISMA 2020 flow diagram of the study selection process for the scoping review. (DOCX)

## References

[1] Global report on hypertension_ the race against a silent killer - World Health Organization - Google Books.

[2] Tan J, Thakur K, Liang Tan J. Hypertension, Systolic [Internet]. Available from: https://www.researchgate.net/publication/323509282

[3] Oti-Boadi E, Osei EA, Asare B, Ampong S, Asiedu PO, Hakami FA, et al. Antihypertensive use insights and experiences among hypertensive patients at Korle-Bu teaching hospital. PLoS One. 2024;19(6):e0298202. doi: 10.1371/journal.pone.0298202 38865338 PMC11168662

[4] Adeloye D, Basquill C. Estimating the prevalence and awareness rates of hypertension in Africa: a systematic analysis. PLoS One. 2014;9(8):e104300. doi: 10.1371/journal.pone.0104300 25090232 PMC4121276

[5] Atibila F, Hoor Gt, Donkoh ET, Wahab AI, Kok G. Prevalence of hypertension in Ghanaian society: a systematic review, meta-analysis, and GRADE assessment. Syst Rev. 2021;10(1). 10.1186/s13643-021-01770-x PMC8349493 34364395

[6] Dai B, Addai-Dansoh S, Nutakor JA, Osei-Kwakye J, Larnyo E, Oppong S. The prevalence of hypertension and its associated risk factors among older adults in Ghana. Front Cardiovasc Med. 2022;9. 10.3389/fcvm.2022.990616 PMC9807661 36606290

[7] Olowoyo P, Okekunle AP, Asowata OJ, Atolani S, Morsy MI, Caiazzo E, et al. Prevalence of hypertension in Africa in the last two decades: systematic review and meta-analysis. Cardiovasc Res. 2025;121(12):1815–29. doi: 10.1093/cvr/cvaf125 40662276 PMC12551393

[8] Jeemon P, Séverin T, Amodeo C, Balabanova D, Campbell NRC, Gaita D. World heart federation roadmap for hypertension – A 2021 update. Global Heart. 2021;16. 10.5334/gh.1066 PMC8447967 34692387

[9] mHealth: use of mobile wireless technologies for public health Report by the Secretariat [Internet]. Available from: http://www.who.int/goe/policies/en

[10] Free C, Phillips G, Watson L, Galli L, Felix L, Edwards P, et al. The effectiveness of mobile-health technologies to improve health care service delivery processes: a systematic review and meta-analysis. PLoS Med. 2013;10(1):e1001363. doi: 10.1371/journal.pmed.1001363 23458994 PMC3566926

[11] Jones LM, Monroe KE, Tripathi P, Bashshur MJ, Kavalakatt J, Tarrance K. Empowering WHISE women: usability testing of a mobile application to enhance blood pressure control. Mhealth. 2024;10. 10.21037/mhealth-24-6 PMC11304098 39114460

[12] Arshed M, Mahmud AB, Minhat HS, Ying LP, Umer MF. Effectiveness of mHealth Interventions in Medication Adherence among Patients with Cardiovascular Diseases: A Systematic Review. Diseases. 2023;11. 10.3390/diseases11010041 PMC10047328 36975590

[13] Lu X, Yang H, Xia X, Lu X, Lin J, Liu F, et al. Interactive mobile health intervention and blood pressure management in adults: A meta-analysis of randomized controlled trials. Hypertension. 2019;74(3):697–704. 31327259 10.1161/HYPERTENSIONAHA.119.13273

[14] Dele-Ojo BF, Oseni TIA, Duodu F, Echieh CP, Blankson PK, Alabi BS. The effect of mobile health technology on blood pressure control among patients with hypertension in Ghana and Nigeria. Res Sq. 2023.

[15] Ataklte F, Erqou S, Kaptoge S, Taye B, Echouffo-Tcheugui JB, Kengne AP. Burden of undiagnosed hypertension in sub-saharan africa: A systematic review and meta-analysis. Hypertension. 2015;65(2):291–8. 25385758 10.1161/HYPERTENSIONAHA.114.04394

[16] Ansu-Mensah M, Mohammed T, Udoh RH, Bawontuo V, Kuupiel D. Mapping evidence of free maternal healthcare financing and quality of care in sub-saharan africa: A systematic scoping review protocol. Health Res Policy Syst. 2019;17(1). 10.1186/s12961-019-0495-1 PMC6882201 31775899

[17] Hoffer-Hawlik M, Moran A, Zerihun L, Usseglio J, Cohn J, Gupta R. Telemedicine interventions for hypertension management in low- and middle-income countries: A scoping review. PLoS ONE. 2021;16. 10.1371/journal.pone.0254222 PMC8270399 34242327

[18] McLean G, Band R, Saunderson K, Hanlon P, Murray E, Little P, et al. Digital interventions to promote self-management in adults with hypertension systematic review and meta-analysis. J Hypertens. 2016;34(4):600–12. doi: 10.1097/HJH.0000000000000859 26845284 PMC4947544

[19] Levac D, Colquhoun H, O’Brien KK. Scoping studies: advancing the methodology. Implement Sci. 2010;5:69. doi: 10.1186/1748-5908-5-69 20854677 PMC2954944

[20] Dlamini SB, Sartorius B, Ginindza T. Mapping the evidence on interventions to raise awareness on lung cancer in resource poor settings: A scoping review protocol. Syst Rev. 2019;8(1). 10.1186/s13643-019-1138-x PMC6708127 31445512

[21] Tricco AC, Lillie E, Zarin W, O’Brien KK, Colquhoun H, Levac D, et al. PRISMA Extension for Scoping Reviews (PRISMA-ScR): Checklist and Explanation. Ann Intern Med. 2018;169(7):467–73. doi: 10.7326/M18-0850 30178033

[22] Braun V, Clarke V. Using thematic analysis in psychology. Qual Res Psychol. 2006;3(2):77–101.

[23] Widmer RJ, Collins NM, Collins CS, West CP, Lerman LO, Lerman A. Digital health interventions for the prevention of cardiovascular disease: a systematic review and meta-analysis. Mayo Clin Proc. 2015;90(4):469–80. 25841251 10.1016/j.mayocp.2014.12.026 PMC4551455

[24] Gong K, Yan Y-L, Li Y, Du J, Wang J, Han Y, et al. Mobile health applications for the management of primary hypertension: A multicenter, randomized, controlled trial. Medicine (Baltimore). 2020;99(16):e19715. doi: 10.1097/MD.0000000000019715 32311957 PMC7440290

[25] Wang J, Wang Y, Wei C, Yao NA, Yuan A, Shan Y, et al. Smartphone interventions for long-term health management of chronic diseases: an integrative review. Telemed J E Health. 2014;20(6):570–83. doi: 10.1089/tmj.2013.0243 24787747

[26] Hurt K, Walker RJ, Campbell JA, Egede LE. mHealth Interventions in Low and Middle-Income Countries: A Systematic Review. Glob J Health Sci. 2016;8(9):54429. doi: 10.5539/gjhs.v8n9p183 27157176 PMC5064069

[27] Hensher M, Cooper P, Dona SWA, Angeles MR, Nguyen D, Heynsbergh N, et al. Scoping review: Development and assessment of evaluation frameworks of mobile health apps for recommendations to consumers. J Am Med Inform Assoc. 2021;28(6):1318–29. doi: 10.1093/jamia/ocab041 33787894 PMC8263081

[28] Cao W, Milks MW, Liu X, Gregory ME, Addison D, Zhang P. mHealth interventions for self-management of hypertension: Framework and systematic review on engagement, interactivity, and tailoring. JMIR mHealth and uHealth. 2022;10. 10.2196/29415 PMC8928043 35234655

