# Supplementary material for: Implementation of mobile health interventions in hypertension management and outcomes: A scoping review protocol
Source: PLoS One. 2026 Feb 5;21(2):e0342224. doi: 10.1371/journal.pone.0342224 (PMC12875436; doi:10.1371/journal.pone.0342224)
Supplement: S1 Table — (DOCX) [file pone.0342224.s001.docx]

**S1_Table: Study eligibility criteria used for selecting articles in the scoping review.**

| **Eligibility criteria** | **Include** | **Exclude** |
| --- | --- | --- |
| **Population** | - Studies involving adult patients (18 years and above) diagnosed with hypertension, regardless of gender, ethnicity, or geographic location. - Studies involving healthcare providers or caregivers using mHealth interventions to manage hypertension. | - Studies focusing on children, adolescents (under 18), or populations with other non-communicable diseases (e.g., diabetes, heart failure, or kidney disease) - Studies in which patients have multiple conditions and the intervention does not specifically target hypertension alone. - Studies where hypertension is only mentioned as a background characteristic, but the intervention focuses on a different condition |
| **Concept/Intervention** | - Studies that describe or evaluate the implementation of mHealth interventions aimed at managing hypertension. These mHealth interventions will include: mobile applications (apps) (e.g., smartphone apps for blood pressure monitoring, medication reminders, or lifestyle changes); SMS (text message)-based interventions; wearable devices linked to mobile devices; and telemedicine platforms focused on hypertension management. | - Studies focusing on non-digital interventions (e.g., traditional face-to-face health education or counselling or paper-based education materials). - Studies evaluating general health apps not specifically aimed at managing hypertension. |
| **Context** | - Studies reporting on any of the following health outcomes or implementation outcomes related to mHealth interventions for hypertension management: Blood pressure control or reduction; medication adherence; lifestyle changes (e.g., diet, physical activity); patient engagement or satisfaction; health-related quality of life; and implementation outcomes (e.g., feasibility, acceptability, sustainability, scalability) - A clear intervention timeline outlining key phases such as app development, stakeholder consultation, pilot deployment, and training rollout. | - Studies that do not report any relevant health or implementation outcomes related to hypertension management. - Studies that do not report a clear intervention timeline on App development, stakeholder consultation. Pilot deployment and training rollout. |
| **Setting** | - Studies conducted in healthcare settings, community settings, or home environments. | - Studies targeting individuals without hypertension or not focusing on blood pressure management, regardless of the setting. |
| **Study design** | - Primary empirical studies, including randomized controlled trials, quasi-experimental studies, observational studies, qualitative studies, and mixed-methods studies examining mHealth interventions for hypertension management. | - Systematic reviews, scoping reviews, meta-analyses, narrative reviews, editorials, commentaries, and opinion pieces. |
| **Publication type and language** | - Peer-reviewed articles (e.g., reports, theses) in English. | - Non-peer reviewed publications like Thesis, dissertation, reports, preprints. |
| **Publication language** | - English. | - Studies published in languages other than English, unless translation is available. |
| **Date/Time** | - Studies published from 2014 to 2025. Studies will not be limited by region-specific time zones. | - Studies published prior to 2014 will be excluded. |
