# Supplementary material for: Implementation of mobile health interventions in hypertension management and outcomes: A scoping review protocol
Source: PLoS One. 2026 Feb 5;21(2):e0342224. doi: 10.1371/journal.pone.0342224 (PMC12875436; doi:10.1371/journal.pone.0342224)
Supplement: S2 Table — (DOCX) [file pone.0342224.s002.docx]

**S2_Table: PubMed database pilot search strategy used to inform development of the final search strategy for the scoping review.**

| **Date** | **Database** | **Keywords** | **Search Result** |
| --- | --- | --- | --- |
| 08/09/2024 | PubMed | Search # 1: (hypertension OR “high blood pressure” OR BP) AND (“home-based management” OR self-monitoring) | 128 |
|  |  | Search # 2: (hypertension OR “high blood pressure”) AND (mHealth OR “mobile health” OR eHealth OR telemedicine) AND (strategy* OR digital intervention*) | 187 |
|  |  | Search # 3: (hypertension AND patient) AND (“mobile health” OR mHealth OR “telemedicine” OR “remote care” OR eHealth) AND (management OR outcome) | 165 |
|  |  | Search # 4: (hypertension AND (mHealth OR “mobile health” OR eHealth OR telemedicine)) AND (“health outcome” OR effectiveness) | 158 |
|  |  | Search # 5: (hypertension OR “high blood pressure”) AND (“digital health” OR mHealth OR eHealth OR telemedicine) AND (“blood pressure management” OR control) | 183 |
|  |  | Search # 6: (hypertension OR “high blood pressure”) AND (“mobile health intervention” OR mHealth OR eHealth OR “digital intervention”) | 230 |
|  |  | Search # 7: ((hypertension OR “blood pressure control”) AND (mHealth OR “mobile health” OR telemedicine OR eHealth)) | 240 |
|  |  | Search # 8: (hypertension AND (“digital health” OR mHealth OR “mobile health” OR telemedicine OR eHealth)) | 256 |
|  |  | Search # 9: ((hypertension OR mHealth OR “mobile health” OR telemedicine OR eHealth) AND (“manage hypertension” OR management)) | 4313 |
|  |  | Search # 10: (hypertension OR “high blood pressure” OR BP) AND (“mobile health” OR mHealth OR smartphone OR “mobile application” OR app-based OR digital health OR eHealth OR telemedicine OR telehealth) AND (“blood pressure control” OR “medication adherence” OR self-monitoring OR “home monitoring” OR patient management OR “digital intervention”) | 245 |
