# Supplementary material for: Implementation of mobile health interventions in hypertension management and outcomes: A scoping review protocol
Source: PLoS One. 2026 Feb 5;21(2):e0342224. doi: 10.1371/journal.pone.0342224 (PMC12875436; doi:10.1371/journal.pone.0342224)
Supplement: S1 Fig — (DOCX) [file pone.0342224.s004.docx]

**Identification**

Records screened

(n = )

Reports sought for retrieval

(n = )

Reports assessed for eligibility

(n = )

Reports excluded:

Reason 1 (n = )

Reason 2 (n = )

Studies included in review

(n = )

Reports of included studies

(n = )

**Screening**

**Included**

Records identified from*:

Databases (n = )

Records removed *before screening*:

Duplicate records removed (n = )

Records marked as ineligible by automation tools (n = )

Records removed for other reasons (n = )

Records excluded**

(n = )

Reports not retrieved

(n = )

Records identified from:

Websites (n = )

Organisations (n = )

Citation searching (n = )

etc.

Reports assessed for eligibility (n = )

Reports excluded:

Reason 1 (n = )

Reason 2 (n = )

Reports sought for retrieval

(n = )

Reports not retrieved

(n = )

**S1_Fig. PRISMA 2020 flow diagram of the study selection process for the scoping review.**
